# Production and perception of contrast: The case of the rise-fall contour in German

**DOI:** 10.3389/fpsyg.2015.01254

**Published:** 2015-09-02

**Authors:** Frank Kügler, Anja Gollrad

**Affiliations:** Department Linguistik, Universität PotsdamPotsdam, Germany

**Keywords:** production of contrast, perception of contrast, semantic-congruency task, rise-fall contour, German intonation

## Abstract

This study investigates the phonetics of German nuclear rise-fall contours in relation to contexts that trigger either a contrastive or a non-contrastive interpretation in the answer. A rise-fall contour can be conceived of a tonal sequence of L-H-L. A production study elicited target sentences in contrastive and non-contrastive contexts. The majority of cases realized showed a nuclear rise-fall contour. The acoustic analysis of these contours revealed a significant effect of contrastiveness on the height/alignment of the accent peak as a function of focus context. On the other hand, the height/alignment of the low turning point at the beginning of the rise did not show an effect of contrastiveness. In a series of semantic congruency perception tests participants judged the congruency of congruent and incongruent context-stimulus pairs based on three different sets of stimuli: (i) original data, (ii) manipulation of accent peak, and (iii) manipulation of the leading low. Listeners distinguished nuclear rise-fall contours as a function of focus context (Experiment 1 and 2), however not based on manipulations of the leading low (Experiment 3). The results suggest that the alignment and scaling of the accentual peak are sufficient to license a contrastive interpretation of a nuclear rise-fall contour, leaving the rising part as a phonetic onglide, or as a low tone that does not interact with the contrastivity of the context.

## 1. Introduction

This paper reports the results of a production experiment and a series of perception experiments that concern the prosodic expression of contrast in German. In particular, we investigate the phonetic details of the rise-fall contour in contexts that license either a non-contrastive or contrastive interpretation of the answer. The perception experiments seek to clarify the functional interpretation of the rise-fall contour in these contexts. In the following section a brief background on the focus-to-accent theory and the theory of intonational meaning is provided, which is mostly based on a discussion of English intonation. This discussion is followed by a brief review of German intonation and its relation to the prosodic expression of focus and contrast.

### 1.1. Focus-to-accent theory and intonational meaning

Focus-to-accent theory proposes that the semantic interpretation of a focus in a sentence is distinguished from its phonological interpretation by means of the presence of a pitch accent (Gussenhoven, 1984; Selkirk, 1984). Hence, focus defined as an indication of “the presence of alternatives that are relevant for the interpretation of linguistic expressions” (Krifka, 2008, p. 247) represents an abstract cognitive category which is prosodically expressed in language-specific ways. Syntactically, it is assumed that the focused constituent is F-marked (Jackendoff, 1972; Gussenhoven, 1984; Selkirk, 1984; Truckenbrodt, 2012). The presence of an F-mark is then assumed to have certain, language-specific effects on the phonological and phonetic expression of the focussed constituent. For instance, consider (1). While the context in (1-a) licenses the whole sentence as one of the alternatives, the context in (1-b) licenses only one particular constituent, i.e., the whale, as an alternative. The difference in F-marking in (1) then is expected to show a difference in the prosodic realization of the answer.

**Table:** 

(1)	a.	A: Erzähl mir bitte, was passiert ist. ‘Please tell me, what happened?’
		B: [ Martin hat den Wal gesehen. ]_*F*_ ‘Martin has seen the whale.’
	b.	A: Hat Martin den Frosch gesehen? ‘Has Martin seen the frog?’
		B: Nein. Martin hat den [ Wal ]_*F*_ gesehen. ‘Martin has seen the whale.’

The concept of contrast in linguistic research has a long research tradition and is generally connected to information structural categories such as topic or focus. Whether “contrast” forms its independent category in information structure (Molnár, 2002) or whether it is accompanied with either topic or focus, e.g., (Büring, 2007), remains a debate in linguistics. For an overview on this issue see (Repp, 2010). In this paper, contrast is taken in its pragmatic use for cases where it accompanies focus and corrects a given alternative from an open set of focus alternatives (cf. Krifka, 2008, for the notion of focus and corrective focus).

It is assumed that intonation may, depending on the language and the melody parts, carry post-lexical, sentence-level meaning (Ladd, 2008). In a compositional approach, intonational tones and their combination carry a particular meaning that a speaker may want to convey (Pierrehumbert and Hirschberg, 1990). In particular, Pierrehumbert and Hirschberg (1990) claim that the English pitch accent L+H^*^ carries contrastive meaning while a simple H^*^ pitch accents conveys the meaning of providing new information.

The effect of the two theories, focus-to-accent theory and the theory of intonational meaning, is that a pitch accent carrying a particular meaning has a preference to occur with a context that triggers this particular meaning. In other words, a L+H^*^ pitch accent carrying the contrastive meaning may occur more likely with a context question (1-b) that requires a contrastive interpretation of a constituent in the answer. On the other hand, speakers may produce a H^*^ pitch accent that carries the meaning of providing new information more likely with a context question that requires new information (1-a). Speakers may however vary their prosodic realizations since a context question may allow for different possible answers. Assume for instance that a speaker may imagine that an actual answer in (1-a) is to be contrasted with another conceivable answer. Hence, a speaker may choose, in relation to imaginated additional assumptions about the context, that a contrastive contour may nevertheless be used.

### 1.2. German intonation

The previous discussion was based on English intonation and its analysis of intonational meaning. German intonation differs from English in some respects, yet there are similar assumptions related to the meaning of H^*^ and L+H^*^ pitch accents (Grice et al., 2005). German intonation has been modeled within a number of different frameworks, e.g., in the British School approach to intonation (Klinghardt, 1925, 1927; von Essen, 1964; Pheby, 1975), in terms of different F0 peak alignments (Gartenberg and Panzlaff-Reuter, 1991; Kohler, 1991; Niebuhr, 2007), and in terms of the autosegmental-metrical approach to intonation (Uhmann, 1991; Féry, 1993; Mayer, 1997; Grabe, 1998; Barker, 2002; Braun, 2005; Gilles, 2005; Grice et al., 2005; Peters, 2005, 2006, 2014; Truckenbrodt, 2005, 2007; Baumann, 2006; Kügler, 2007; Bergmann, 2008). Related to the present discussion, work concerning F0 peak alignment has shown that the alignment of accentual peaks is related to the interpretation of information structure categories: an early peak is realized in case of given information, and a late peak in case of focused or new information (Kohler, 1991; Niebuhr, 2007).

As discussed above for English intonation (Pierrehumbert and Hirschberg, 1990), the GToBI system proposes a similar distinction in meaning between H^*^ and L+H^*^ pitch accents in German (Grice and Baumann, 2002; Grice et al., 2005). A nuclear rise-fall contour consists phonologically of a L+H^*^ pitch accent followed by a low phrase accent (L-), cf. (2), and the L+H^*^ pitch accent is assumed to carry contrastive meaning. On the other hand, a plain H^*^ accent is assumed to carry the meaning of newness and thus occurs preferred in non-contrastive contexts. Data on the frequency of occurrence and distribution of pitch accents in different contexts support the outlined preferences (Baumann et al., 2006; Grice et al., 2009; Sudhoff, 2010).

**Table:** 

(2)	a.	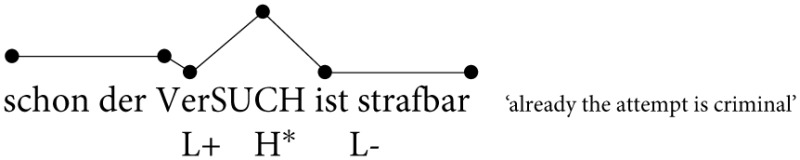
	b.	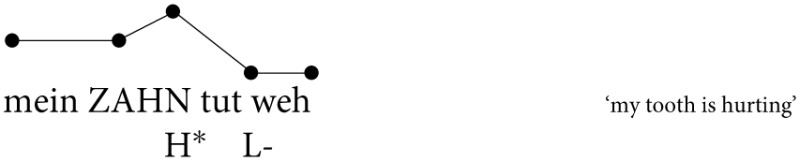

According to Féry (1993), however, there is no phonological distinction between a pitch accent realized under contrastive and broad focus in German. Hence, the accent shapes as in (2) are analyzed with a falling H^*^+L pitch accent independent of a contrastive or non-contrastive context (cf. also Grabe, 1998; Kügler et al., 2003; Peters, 2005, 2006, 2014). The varying accent shapes illustrated in (2) are all taken to constitute a rise-fall contour. Both Féry (1993) and Grabe (1998) claim that in case of a nuclear rise-fall contour the pitch rise toward the pitch peak is phonetic in nature. The assumption is that the tonal grammar of German does not exhibit a L+H^*^ pitch accent (Féry, 1993; Grabe, 1998).

The prosodic realization of contrast in German has been intensively studied. Generally, a focus is prosodically marked by means of a pitch accent in German (Uhmann, 1991; Féry, 1993; Grice et al., 2009), except for particular cases of secondary focus (Féry and Ishihara, 2009; Baumann et al., 2010). Some researchers argue that slight phonetic differences between non-contrasted and contrasted realizations such as greater intensity and F0 excursion are neither necessary nor sufficient cues to signal contrast in German (Fuchs, 1976). The majority of studies however show clear and distinct differences in the production of pitch accents in non-contrasted as opposed to contrasted contexts in German (Bannert, 1985; Alter et al., 2001; Braun, 2005, 2006; Baumann et al., 2006, 2007; Féry and Kügler, 2008; Kügler, 2008; Grice et al., 2009; Sudhoff, 2010). Although the studies differ slightly in the number and kind of phonetic cues that are expressed as an interpretation of contrastiveness, generally, greater F0 excursion, or higher F0 maximum and lower F0 minimum, longer duration of the accented syllable as well as higher intensity are listed to be the relevant prosodic correlates that signal contrast in German.

This difference in prosodic marking of contrast has led to a number of studies investigating the concept of contrast in psycholinguistic research (e.g., Alter et al., 2001; Carlson, 2001; Toepel et al., 2005). As for German, the studies disagree whether or not the phonetic cues associated with contrastive accents necessarily have to be correlated with a different phonological category. Even though the prosodic cues that signal contrast in German are used to study parsing effects of ambiguous clauses, the phonological analysis of accents in contrastive contexts is still a matter of debate. Baumann et al. (2006), Braun (2006), Grice et al. (2009), and Sudhoff (2010) show that there is a considerable amount of speaker variation with respect to which pitch accent type is used in contrastive contexts compared to a neutral accentuation. In particular, Baumann et al. (2006) show that in neutral accentuation speakers tend to use downstepped accents more frequently than in case of focus, be it narrow information focus or contrastive focus (cf. also Féry and Kügler, 2008, for the preference of downstepped accents in broad focus contexts over contrastive focus contexts). However, some speakers in their study only use one identical high pitch accent independent of focus structure. The issue of speaker variation is not investigated in the current study since we are concentrating on a particular type of nuclear contour and its functional property to signal contrast.

Previous results indicate some degree of free variation with respect to accent realization, and our results of the production data show that not all speakers use raised accentual peaks in order to signal contrast. While some researchers assume a phonological difference between L+H^*^ and H^*^ pitch accents and their accompanied difference in meaning that these accents express (e.g., Grice et al., 2005; Baumann et al., 2006; Sudhoff, 2010), other researchers claim that focus and/or contrast are prosodically expressed by means of pitch register changes (e.g., Féry and Kügler, 2008; Féry and Ishihara, 2010) thus not postulating a phonological distinct representation with distinct meanings.

In order to study the prosodic expression of contrast in German, the rise-fall contour is particularly suitable since the rise can be attributed to the L+H^*^ pitch accent which is assumed to carry the meaning of contrast (cf. Grice et al., 2005). On the other hand, a rise-fall contour may be realized in a broad focus context according to Féry (1993). This study will therefore examine the phonetics of the rise-fall contour in German. The contours illustrated in (2) are assumed to constitute variants of the rise-fall contour, and we used contexts that either elicited a contrastive or a non-contrastive interpretation of a particular constituent in the answer. The first question to be explored is whether speakers produce a systematic difference between rise-fall contours as a function of different contexts. The second question is whether perception tests reveal which parts of the rise-fall contour carry a functional interpretation of contrast. The next section briefly introduces methods for testing the perception of intonation.

### 1.3. Methods for testing intonational categories

In intonation research a considerable body of research is concerned with the investigation of the appropriate method to test intonational categories perceptually (Gussenhoven, 1999). Different methods such as identification and discrimination studies within the categorical perception paradigm (Kohler, 1987; Gartenberg and Panzlaff-Reuter, 1991; Ladd and Morton, 1997; Remijsen and van Heuven, 1999; Post, 2000; Schneider and Lintfert, 2003; Niebuhr and Kohler, 2004; Cummins et al., 2006), imitation studies (Pierrehumbert and Steele, 1989; Redi, 2003; Dilley, 2005; Dilley and Brown, 2007; Dilley, 2010), the gating paradigm (Petrone and Niebuhr, 2014), and/or prominence judgments or semantic scales (Rietveld and Gussenhoven, 1985; Gussenhoven and Rietveld, 1988; Ladd et al., 1994) have been used and showed different success, for an overview of current methods see (Prieto, 2012).

In recent years, however, researchers emphasize the role of functional perception tests (Prieto, 2012) for the identification of tonal categories since the intonation carries function and meaning. In particular, semantic judgments were employed to test the function and meaning of intonational categories (Nash and Mulac, 1980; Gussenhoven and Rietveld, 2000; Niebuhr, 2007). Semantic congruency tests were used to study tonal categories in its appropriate context (Rathcke and Harrington, 2010; Kügler and Gollrad, 2011; del Mar Vanrell et al., 2013). The present study relies on the method of semantic congruency to test the function and meaning of the rise-fall contour in its context.

## 2. Speech production experiment

### 2.1. Method

#### 2.1.1. Speech materials

The speech production experiment examines the prosodic realizations of broad and contrastive focused sentences by comparing the phonetics of the nuclear rise-fall contour in German. The experimental sentences contain the word order subject-auxiliary-object-verb (SAuxOV). The target words were embedded as objects in non-final sentence position in order to avoid any intonational phrase boundary effects. The following two factors were manipulated in order to elicit a nuclear rise-fall contour:

- The number of syllables ofx the target word varied between one (Wal [vaːl] “whale”), two (Roman [ro.ˈmaːn] “novel”), and three (Admiral [ad.mi.ˈraːl] “admiral”), all with ultima word stress. Ultima word stress was chosen to provide segmental space for a low leading tone within the accented word if such a category exists. Word-level effects on tonal alignment have been shown for English (Ladd and Schepman, 2003).
- The length of the sentence: From the basic SAuxOV structure, sentences were gradually lengthened by adding one of the two adverbials (gestern “yesterday”), and (glücklicherweise “luckily”) or a combination of both prior to the target word to increase the interaccentual distance between a prenuclear, sentence initial accent, and the nuclear accent on the target word. We expected that a larger interaccentual distance would increase the chance that speakers realize two single peak accents (Kügler et al., 2003) instead of a hat pattern, which is a frequent pattern in German (Féry, 1993; Braun, 2006). Stretching the interaccentual distance between prenuclear and nuclear accents within a sentence was not thought of being an independent factor influencing the phonetic characteristics of nuclear accents as such but was rather a strategy to ensure a large data set for the phonetic measurements. The entire material used in the production experiment is listed in the Supplementary Material.

As an experimental factor, Focus was manipulated eliciting broad and contrastive focus. (3-a) illustrates a context that elicits the broad focus target sentence (3-b). (4-a) illustrates a context that elicits a sentence with a contrastively focused target word (4-b). In both examples, the target word is monosyllabic.

**Table:** 

(3)	a.	Erzähl mir bitte, was passiert ist. ‘Please tell me what happened.’
	b.	Maja hat den Hahn gefüttert. Maja has the cock fed ‘Maja has fed the cock.’
(4)	a.	Hat Maja den Hund gefüttert? ‘Has Maja fed the dog?’
	b.	Nein, Maja hat den Hahn gefüttert. No, Maja has the cock fed ‘No, Maja has fed the cock.’

The experimental sentences are highly sonorant to allow for a maximally accurate F0 analysis. Sentences were interspersed with fillers (proportion of target-filler sentences was 1:3) and fed into the DMDX presentation software (Forster and Forster, 2003). The experimental sentences were pseudorandomized for each subject so that sentences of the same condition did not appear adjacently and corresponding sentences had a maximal distance.

#### 2.1.2. Speakers

Eight speakers participated in the experiment. All were female undergraduate students at the University of Potsdam in their twenties. All were native speakers of standard German spoken in the Berlin-Brandenburg region and reported no speech or hearing impairment. They either received course credit or were paid for participation. All subjects of this production study and of subsequent perception experiments gave written informed consent in accordance with the Declaration of Helsinki.

#### 2.1.3. Recording procedure

For each sentence, a context eliciting broad focus (3-a) and contrastive focus (4-a), spoken by a male voice, had been previously recorded. The contexts were presented together with a target sentence both visually on screen and auditorily over headphones. The pre-recorded context sentences ensured that no uncontrolled variation of an experimenter speaking the context questions would affect the data elicitation. Speakers were asked to read and listen to the context and then to speak out the answer displayed on the screen as a response to the question. Subjects were familiarized with the task through written and verbal instructions. In case of hesitations or false starts, participants were asked to repeat the sentence. Recordings took place in a sound-proof chamber equipped with an AT4033a audiotechnica studio microphone, using a C-Media Wave sound card at a sampling rate of 44.1 kHz with 16 bit resolution. Presentation flow was controlled by the experimenter, and participants were allowed to take a break at any point. A total of 384 target sentences (8 speakers × 2 focus conditions × 6 target words × 4 sentence lengths) had been recorded.

#### 2.1.4. Grouping of nuclear contours

**Figure 1:**
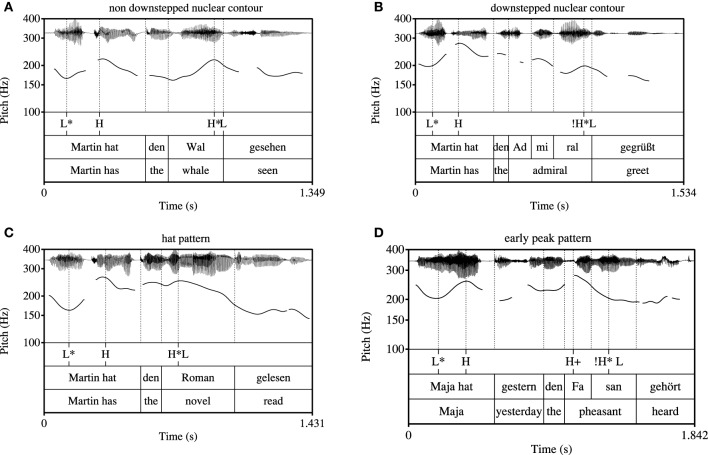
**Pitch track representation of the four phonological contours, from top left to bottom right: (A) non downstepped nuclear contour, (B) downstepped nuclear contour, (C) hat pattern, and (D) early peak pattern**. All four contours involve a prenuclear accent followed by a nuclear accent. The target word carries the nuclear accent, which is associated with the most prominent syllable.

As there is a range of possible nuclear intonation contours in German (Féry, 1993; Grabe, 1998; Grice et al., 2005, 2009), we grouped nuclear contours according to their overall shape. Since we are interested in the nuclear rise-fall contour, we separated these from other nuclear contours. We established four different nuclear contours in our data which are illustrated in Figure 1. The annotation of the pitch contours was based on the tonal grammar of German proposed by Féry (1993). The total of 384 sentences was subgrouped into the four distinct phonological contours as follows:

Subgroup (a) contains 255 non-downstepped nuclear rise-fall contours, which comprise contours that contain either a prenuclear rising or falling accent (cf. Uhmann, 1991; Féry, 1993). Figure 1A illustrates a nuclear rise-fall contour with a prenuclear rising accent. The two accents in Figure 1A have comparable F0 scaling concerning their H tones. The instances of the rise-fall contour constitute the cases for further phonetic analysis.

Subgroup (b) contains 25 downstepped nuclear rise-fall contours, which comprise either rising or falling prenuclear accents. The H tone of the nuclear accent in Figure 1B is scaled lower relative to its preceding H tone of the prenuclear accent, which causes the perceptual impression of a downstep. The downstep is indicated by the exclamation mark. Note that downstepped accents lack clear low turning points in F0 prior to the downstepped peak in most of the cases. Nuclear downstepped accents are used frequently in German (Féry and Kügler, 2008; Grice et al., 2009).

The third subgroup (c) consist of 36 hat patters (Kohler, 1991; Uhmann, 1991; Féry, 1993; Braun, 2006) (cf. “bridge accent” in Wunderlich, 1988), which results from a prenuclear rising or high pitch accent and a nuclear falling pitch accent. Both accents are concatenated by a high F0 plateau, without a dip between the prenuclear and the nuclear accent (cf. Figure 1C).

The fourth subgroup (d) contains 68 other types of nuclear accents, such as early peaks. This category displays cases of a prenuclear accent followed by a nuclear accent, where the nuclear accent displays a different alignment shape as the ones before. In Figure 1D the peak of the falling accent is aligned with the syllable preceding the stressed syllable of the target word, a case referred to as early peak (Kohler, 1991; Uhmann, 1991; Féry, 1993; Grice et al., 2005).

Both authors conducted the grouping independently and agreed in about 92% of the cases. For the remaining cases, we discussed each individual contour by listening and looking at the F0 contour to eventually decide on the contour.

As can be seen in Table 1, the non-downstepped rise-fall contours are almost equally distributed across the two context conditions. In 45% of the 255 cases, a rise-fall was realized in a broad focus context, somewhat more (55%) in a context eliciting contrastive focus. In these realizations we analyzed how a contrastive focus changes the phonetic realization of the rise-fall. Table 1 also shows that 19 downstepped accents and 29 hat patterns are preferred realizations in a broad focus context (80 and 76%, respectively), which is in line with previous findings (cf. Grice et al., 2009). In the following, group (a) is investigated in more detail.

**Table 1:** **Distribution of nuclear contours per subgroup, split by focus condition**.

**Phonological contour**	**Broad focus**	**Contrastive focus**	***n***
(a) Non-downstepped	115	140	255
(b) Downstepped	19	6	25
(c) Hat pattern	29	7	36
(d) Other (early peak)	29	39	68
Sum	192	192	384

#### 2.1.5. Data processing

**Figure 2:**
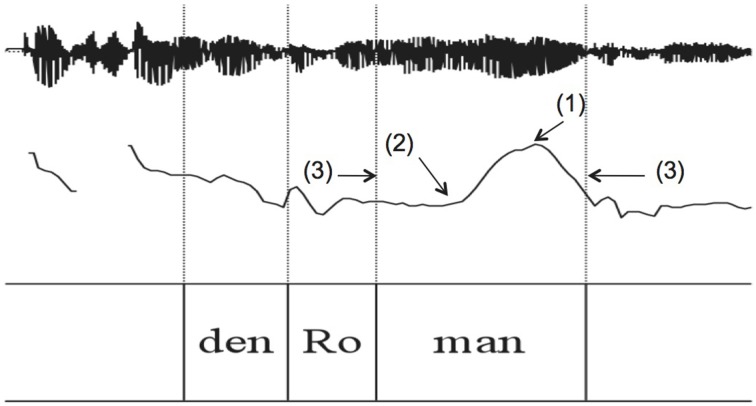
**Phonetic measurements of the target word; for measuring points (1), (2), and (3) see text**.

The 255 experimental sentences of group (a) were hand-annotated and subjected to phonetic analysis using Praat software (Boersma and Weenink, 2013). The annotation comprised the target noun phrase including the determiner, see Figure 2. Annotation was done on the level of the syllable. The following phonetic measurements were conducted, numbers correspond to measuring points in Figure 2:

- The pitch peak (H) of the target words in Hertz (Hz), see point (1) in Figure 2
- The corresponding time of the peak (*t*_*H*_), see point (1) in Figure 2
- A low turning point in pitch prior to the peak (l) in Hz, which corresponds to the “elbow” measure in D'Imperio (2000), see point (2) in Figure 2
- The corresponding time of the low turning point (*t*_*l*_), see point (2) in Figure 2
- The beginning and the end of the accented syllable (*t*_*beg*_, *t*_*end*_), see point (3) in Figure 2

Pitch analysis was conducted using a Hanning window of 0.4 s length with a default 10 ms analysis frame. The pitch contour was smoothed using the Praat smoothing algorithm (frequency band 10 Hz) to diminish microprosodic perturbations. Out of these phonetic measurements, the following variables were calculated:

- The excursion (E) between the low turning point and the peak: *E*[Hz] = *H* − *l*.
- The velocity (V) of the pitch rise: $V\left[Hz∕s\right]=\frac{H-l}{t_{H}-t_{l}}$.
- The relative alignment of the the pitch peak (*A*−*H*) with reference to the end of the accented syllable divided by the accented syllable's duration: $A_{H}\left[\%\right]=\frac{t_{H}-t_{end}}{t_{end}-t_{beg}}*100$.
  The end of the accented syllable was chosen based on the results of Grabe (1998) who showed alignment of H^*^ tones at the right edge of the accented syllable's rime.
- The relative alignment of the low turning point (*A*−*l*) with reference to the beginning of the accented syllable divided by the accented syllable's duration: $A_{l}\left[\%\right]=\frac{t_{l}-t_{beg}}{t_{end}-t_{beg}}*100$.
- The duration (D) of the accented syllable: *D*[ms] = *t*_*end*_−*t*_*beg*_.

#### 2.1.6. Statistical analysis

The results of the phonetic calculation were evaluated against the fixed factor Focus [with the two levels broad focus (BF) and contrastive focus (CF)] using linear mixed models (Bates et al., 2013). The reference level in the models was BF. The models applied crossed random factors speaker and item. Random slopes (Barr et al., 2013) for speakers and items were integrated into the models assuming that differences exist for each speaker's individual pitch range. Backward modeling (Barr et al., 2013) of random slopes for speaker and item was applied, and likelihood ratio tests were run to evaluate the models. The basis for removing factors was a *p*-value of the likelihood ratio test of *p* < 0.05 and lower AIC values.

### 2.2. Results

The statistical results are shown in Table 2. For each individual variable it is shown which model presents the best fit. Significance at the level *p* < 0.05 for a factor was determined with an absolute *t*-value of 2 or greater (Barr et al., 2013). We find a significantly lower excursion size (E) in BF compared to CF (means for BF: 45.7 Hz and CF: 52.2 Hz), a significantly slower velocity of the rise (V) in BF compared to CF (means for BF: 262.6 Hz/s vs. CF: 305.0 Hz/s), a significantly earlier alignment of the accentual peak in relation to the end of the syllable (A–H) in BF compared to CF (means for BF: 20.30% and CF: 11.78%), and a significantly shorter duration (D) in BF compared to CF (means for BF: 247 ms and CF: 261 ms). The model for the scaling of the accentual peak (H) reveals a near significant effect between both focus conditions (means for BF: 236.1 Hz and CF: 240.8 Hz). The analysis reveals that in contrastive contexts the accentual peak is affected. It is realized higher and it occurs later. In absolute values, the low turning point prior to the accentual peak [L (Hz)] does not differ systematically between both focus conditions (means for BF: 190.4 Hz and CF: 188.6 Hz), nor does the relative alignment of the low turning point (A–L) differ between focus conditions (means for BF: 12.39% and CF: 18.94%). The fact that the velocity of the rise and the excursion size show a significant effect is compatible with the change being located only in the H.

**Table 2:** **Report of the linear mixed-effects models for each of the measured cues**.

**Variable**		**Coefficients**	**SE**	***t*-value**	**Sign**.
L (Hz)	Intercept	191.187	6.623	28.439	
	CF	−2.579	2.271	−1.136	n.s.^a^
H (Hz)	Intercept	233.67	8.415	27.769	
	CF	4.728	2.471	1.913	(^*^)^a^
E(Hz)	Intercept	42.416	6.287	6.746	
	CF	7.412	3.524	2.103	^*^^a^
V (Hz/s)	Intercept	251.19	31.52	7.969	
	CF	46.85	11.03	4.247	^*^^b^
A–L (%)	Intercept	14.275	4.568	3.125	
	CF	4.940	4.401	1.122	n.s.^a^
A–H (%)	Intercept	19.98	1.671	11.953	
	CF	−8.31	2.223	−3.738	^*^^a^
D (ms)	Intercept	243.297	8.361	29.10	
	CF	15.581	3.898	3.997	^*^^b^

*^a^ Based on a linear mixed model including item with random intercepts and subjects with random intercepts and random slopes*.

*^b^ Based on a linear mixed model including item and speakers with random intercepts only*.

* *indicates significance at the level p < 0.05; n.s. refers to non-significance*.

### 2.3. Discussion

The analysis of the phonetic variables yields no clear indication that the low F0 turning point prior to the accentual peak represents a systematic difference between the two focus contexts. Neither the model for L-tone scaling nor the model for L-tone alignment showed a systematic difference between a broad and a contrastive context. On the other hand, the model for scaling and the model for alignment of the accentual peak showed differences as a function of focus context. The scaling of the accentual H tone is higher in contrastive focus contexts, which is well in line with previous findings (Bannert, 1985; Alter et al., 2001; Braun, 2005, 2006; Baumann et al., 2006, 2007; Féry and Kügler, 2008; Grice et al., 2009; Sudhoff, 2010); the effect approaches significance. The significantly increased duration is also well-known for German (cf. e.g., Kügler, 2008).

The fact that H-tone scaling only approaches significance seems to be due to the fact that not all speakers employ this strategy to realize contrastive focus. Model comparison for H-tone scaling applying likelihood ratio tests revealed that when removing the slope factor for the random effect of speaker, the effect of Focus on the height of the H becomes significant (*coef* = 2.626, *SE* = 0.841, *t* = 3.122). Thus, the best fit model in Table 2 including the slope effect for speakers indicates speaker-specific differences. We also calculated the individual speaker means which showed that speakers differed considerably in their scaling of the H-tone. Given this finding plus the fact that the model for alignment of the H-tone additionally showed that all speakers employed significantly later accentual peaks in case of contrastive focus contexts suggests that only some speakers employ a different scaling as a means to express contrastive focus. Individual speaker strategies in prosodic focus marking have been reported earlier for German (Baumann et al., 2006). In addition, perception tests showed that the strategy of a higher or later peak revealed identical effects of signaling increased prosodic prominence (Ladd and Morton, 1997). Additionally, duration serves as a robust cue to signal prosodic prominence, and we also found a systematic increase in duration in contrastive contexts.

Furthermore, the phonetic effects triggered by focus should be seen in relation to prenuclear accents. The utterances realized under broad focus exhibit a F0-lowering from prenuclear to nuclear accents, while it is the other way around for the utterances realized under contrastive focus (see Table 3). Paired-samples *t*-tests for broad focus and contrastive focus show that the scaling of prenuclear and nuclear high tones differs significantly. Similar patterns of a relational scaling of pitch accents are reported in Féry and Kügler (2008).

**Table 3:** **Mean F0 maximum in Hz of the prenuclear and nuclear accents, split by focus condition**.

**Focus condition**	**Prenuclear accent**	**Nuclear accent**	**Welch two sample *t*-test**
Broad focus	263.1	236.1	*t* = 5.7551, *df* = 188.384, *p* < 0.001
Contrastive focus	234.0	240.8	*t* = −2.0813, *df* = 269.135, *p* < 0.05

Taken together, the results of the production study indicate that speakers realize a phonetic difference in intonation as a function of the focus condition. In the following series of studies we will test which parts of the rise-fall contour interact perceptually with the contrastivity of the context.

## 3. Speech perception experiments

A series of semantic congruency tasks investigate whether German listeners use the phonetic differences shown in the production study to distinguish the rise-fall contour between contexts that elicit broad or contrastive focus. Semantic congruency tests have been successfully used to explore the perception of functional intonation contrasts (Rathcke and Harrington, 2010; Kügler and Gollrad, 2011; Prieto, 2012; del Mar Vanrell et al., 2013). The test allows us to evaluate the degree of perceived appropriateness of target intonation patterns within different pragmatic contexts.

### 3.1. Perception experiment 1: original data

#### 3.1.1. Material

The first experiment investigates whether the acoustic differences found in the production data are perceived as an indicator for the appropriate context they were realized in. Following different perception studies that rely on the speech of one speaker (Kohler, 1991; Niebuhr, 2007; Dilley and Heffner, 2013) stimulus materials were taken from one of the speakers of the production study. To choose from the eight speakers of the production study, we decided to choose a speaker who produced the most prominent difference from the mean value of the low turning point in both focus conditions.

The target sentences correspond to the six SAuxOV sentences from the production study (cf. Supplementary Material). Each one was uttered in broad focus (BF) and contrastive focus contexts (CF) resulting in 12 sentences. The semantic congruency experiment consisted of these 12 target sentences where intonation was congruent with the pragmatic context (6 BF–BF dialogs, 6 CF–CF dialogs), and 12 cross-spliced target sentences where intonation was incongruent with the pragmatic context (6 CF–BF dialogs, 6 BF–CF dialogs). Stimuli were scaled at an intensity of 70 db. Each dialog was presented 3 times which resulted in a total of 72 dialogs per experiment. The stimuli were auditorily presented over headphones with the MFC Praat software (Boersma and Weenink, 2013). Participants were asked to listen to each dialog carefully and then evaluate whether they regard the intonation of the target sentence to the given context as “congruent” or as “incongruent” (by clicking either on the “congruent box” or the “incongruent box” visible on the screen). After written and verbal instructions, a test run of 3 dialogs was carried out before the experiment started. The experiment lasted approximately 20 min.

#### 3.1.2. Participants

Thirty-six participants took part in the experiment (10 male, 26 female). They were all undergraduates in their twenties, reported no speech or hearing deficits, and were naïve with respect to the purpose of the study. They were either paid for participation or received course credits.

#### 3.1.3. Hypothesis

For the factor Congruency we hypothesize that congruent dialogs (BF–BF and CF–CF pairs) are rated more congruent than incongruent dialogs (CF–BF and BF–CF pairs). This hypothesis reflects the fact that the stimuli produced in their original (= congruent) context are assumed to be perceived as fitting well with their context while cross-spliced context-answer stimuli should create an incongruent impression. As for the factor context we assume no particular effect. In other words, both broad and contrastive contexts are assumed to create the same amount of variation in the perceptual impression.

#### 3.1.4. Results

**Figure 3:**
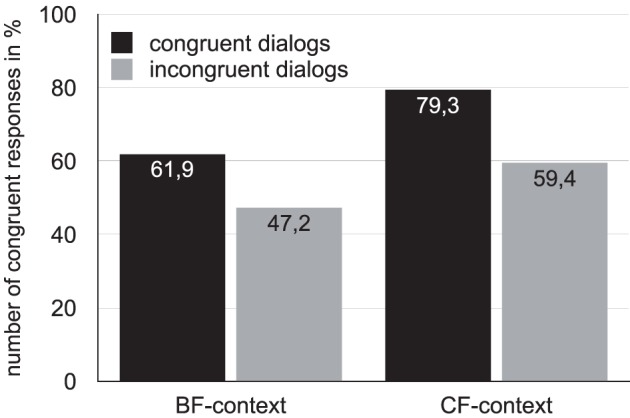
**Number of congruent responses to all four dialog types, separated by context condition**.

Figure 3 displays the rate of congruent responses in percentage to all dialog types, separated into BF-context (left bars) and CF-context (right bars). In general, the appropriateness of the target intonation pattern to a context was rated higher for congruent (BF–BF and CF–CF) than for incongruent dialog types (CF–BF and BF–CF). Specifically, in 61.9% of the BF–BF dialogs, and in 79.3% of the CF–CF dialogs, the target intonation was rated as congruent to its context, while for incongruent dialogs the number of congruent responses was reduced to 47.2% in BF–CF dialogs, and to 59.4% in CF–BF dialogs.

For the statistical, frequency-based analysis, we fit a multilevel model (Bates et al., 2013) using crossed random factors participant and item applying random intercepts and slopes, and context (with levels BF/CF) and congruency (with levels congruent/incongruent) as fixed factors. The analysis relied on the choice of answer (congruent vs. incongruent) as a dependent variable. Treatment-coding was applied using level *BF* of the factor context as baseline, and level *incongruent* of the factor congruency as baseline. Model comparison for the random effect structure was applied, which was based on the same method as described in Section 2.1.6 above.

The model representing the best fit used both random slopes and intercepts of speaker and item for both fixed factors. The model reveals a significant effect for congruency, but neither for context nor for the interaction, cf. Table 4.

**Table 4:** **Report of the linear mixed effects model specified in the text with congruent/incongruent ratings as dependent variable**.

	**Coefficients**	**SE**	***z*-value**	**Sign**.	**Pr(> |*z*|)**
(Intercept)	0.6673	0.1297	5.143	^*^	0.00001
context = *CF*	0.8405	0.5234	1.606	n.s.	0.10833
congruency = *congruent*	0.9649	0.2780	3.470	^*^	0.00052
Interaction	0.2596	0.2651	0.979	n.s.	0.32735

* *indicates significance at level p < 0.05, n.s. refers to non-significance*.

#### 3.1.5. Discussion

The semantic congruency task revealed that listeners judged congruent dialogs as more congruent than incongruent dialogs. The expected effect of congruency was thus borne out. Listeners rely on the phonetic cues in the nuclear rise-fall contour that signal contrastive or non-contrastive interpretations. This result also shows that listeners are able to perceive the subtle acoustic differences that were produced in different contexts. This allows us to continue to investigate which of the acoustic cues, i.e., the accentual high tone or a low turning point in F0, are necessary to perceive the functional difference.

Two subsequent perception experiments were carried out to determine whether the phonetic difference of the high peak or of the low turning point is functionally relevant. The high peak and the low turning point were manipulated separately from each other in two different experiments. The next section describes the phonetic manipulation of the accentual peak on listeners' interpretation in relation to contrast, the third perception experiment investigates the role of the low turning point itself.

### 3.2. Perception experiment 2: manipulation of the H^*^ accent

Given that original stimuli are appropriately categorized according to focus contexts (perception Experiment 1), and in line with previous findings on the effect of contrast on accentual peaks (Ladd and Morton, 1997; Gussenhoven, 2004; Baumann et al., 2006; Féry and Kügler, 2008), we predict that the F0 peak height is functionally relevant, i.e., a higher F0 peak is expected to cause a perceptual impression of contrast.

#### 3.2.1. Speech material

**Figure 4:**
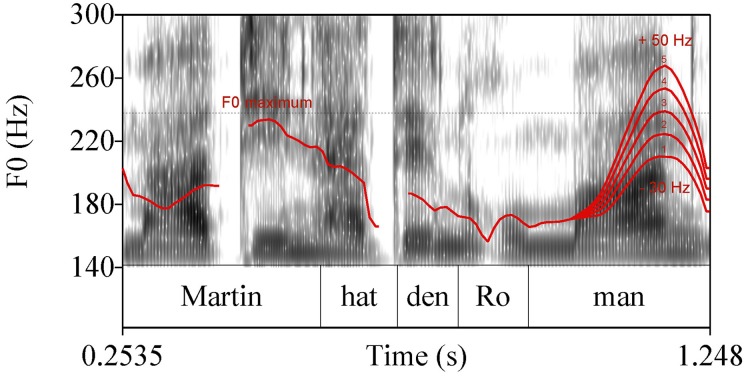
Illustration of the H^*^ manipulation in relation to the prenuclear accent. Numbers 1–5 illustrate the manipulation steps from the lowest H^*^ accent peak (step 1) to the highest H^*^ accent peak (step 5).

We test this prediction by manipulating the scaling of the H^*^ accent successively. The sentences for the H^*^ manipulation were taken from the same speaker used for the first experiment. To keep the total amount of stimuli in a manageable size for a perception study, a total of four target sentences including disyllabic and trisyllabic target words were chosen for the manipulation procedure. These sentences were realized in broad focus contexts, and in contrastive focus contexts yielding eight sentences in total. For each of the 4 sentences, the manipulation of the H^*^ peak was done in relation to the corresponding prenuclear accent on the subject; Figure 4 illustrates this relationship between prenuclear and manipulated nuclear accents. For each sentence, the maximum F0 value on the prenuclear accent was calculated. By adding 50 Hz and by subtracting 30 Hz from the calculated F0 maximum of the prenuclear accent, we defined the manipulation range separately for each sentence. This range corresponds roughly to two standard deviations from the mean F0 value of the nuclear accent peak gained from the production data. The H^*^ accent was manipulated with a Praat script, such that for each original sentence, five stimuli with varying values for the H^*^ peak were re-synthesized; Figure 4 illustrates a horizontal line from the prenuclear peak to the nuclear accent showing two stimuli with lower nuclear peaks, two stimuli with higher nuclear peaks, and one stimulus with identical pitch height compared to the prenuclear accent. Each manipulated target sentence was concatenated with an originally congruent context question (BF–BF, CF–CF) and with an originally incongruent context question (CF–BF, BF–CF), resulting in a total of 80 stimuli (4 sentences × 2 focus conditions × 2 contexts × 5 manipulations). All stimuli were scaled at an intensity of 70 db. Stimuli were subdivided into two lists of 40 stimuli each, such that a participant would hear the same target word originally spoken in one focus condition once in the original matching context and once cross-spliced in the non-matching context. Each list contained 20 congruent and 20 incongruent dialog pairs. The precise grouping arrangement is listed in the Supplementary Material. The reason to divide the stimuli into two lists was to present listeners a comfortable number of dialogs to be evaluated. The experimental task was identical to the one of perception Experiment 1, except that the 80 stimuli were divided into two sets. Participants listened to either set 1 or set 2. The experiment lasted approximately 15 min.

#### 3.2.2. Participants

Forty-eight undergraduate students from Potsdam University (13 male, 35 female) participated in the experiment. They were native speakers of German in their twenties and reported no speech or hearing impairment. The participants were naïve as to the purpose of the experiment and did not participate in perception Experiment 1. Each participant received course credit for participation. Participants were divided into two groups to listen to either the first or the second experimental set.

#### 3.2.3. Hypothesis

If a phonetic cue for contrastiveness (e.g., a higher H^*^), has an effect on the perception of contrast, it will influence the congruency ratings in the two contexts differently: In a contrastive context condition, an effective cue for contrastiveness will lead to more congruency judgements. In a non-contrastive context, an effective cue for contrastiveness will lead to less congruency judgements. For the H^*^ accent manipulation, we expect thus that for contrastive contexts higher F0 peaks (manipulation step 5) cause a perceptual impression of contrastiveness, both in originally congruent (CF–CF) and originally incongruent dialogs (CF–BF) (cf.Baumann et al., 2007; Féry and Kügler, 2008 for higher F0 peaks in German). For broad focus contexts, we expect that lower F0 peaks (manipulation step 1) cause a perceptual impression of broad focus, both in originally congruent (BF–BF) and originally incongruent dialogs (BF–CF); lower peaks are assumed to correspond to the downstep pattern in German broad focus sentences (Féry and Kügler, 2008; Grice et al., 2009). Therefore, we predict that an effective cue for contrastiveness will show an interaction of manipulation and context on the dependent variable congruency.

#### 3.2.4. Results

**Figure 5:**
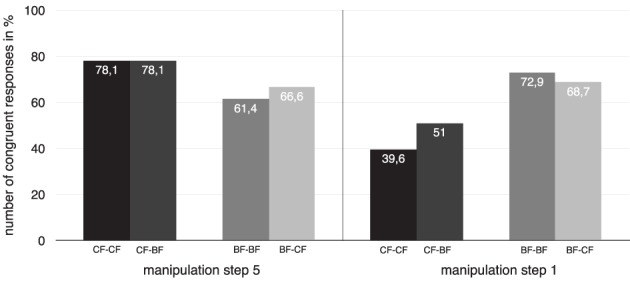
Number of congruent responses to all dialog types, separated by manipulation step 5–highest H^*^ peak (left-hand bars) and manipulation step 1–lowest H^*^ peak (right-hand bars); black and dark gray bars indicate dialog pairs with contrastive contexts, lighter gray bars indicate dialog pairs with broad focus contexts.

Figure 5 displays the results of the H^*^ manipulation experiment separated for the highest H^*^ accent manipulation step 5 (left-hand bars) and the lowest H^*^ accent manipulation step 1 (right-hand bars) for each dialog type. In all contrastive context dialogs under manipulation step 5 (CF–CF and CF–BF), a higher H^*^ accent of the target word leads to a higher number of congruency ratings (both 78.1%) compared to the corresponding dialogs under manipulation step 1 (between 39.6 and 51%). In all broad focus context dialogs under manipulation step 5 (BF–BF and BF–CF), a higher H^*^ accent of the target word leads to approximately identical congruency ratings compared to the corresponding dialogs under manipulation step 1 (ranging between 61.4 and 72.9%).

As described for perception Experiment 1, Section 3.1.4, we fit a multilevel model with context (with levels BF/CF) and manipulation (with levels step1/step5) as fixed factors, and calculated likelihood ratio tests on the basis of backward modeling of the random factors to identify the best fit model. Note that only a subset of the data entered into the analysis, i.e., ratings for the endpoints of the manipulation range, step1 and step5, respectively. This was done to evaluate an effect of the maximal manipulation on the perception; an analysis of the step-wise manipulation is given below. Treatment-coding was applied using level *BF* of factor context, and level *step1* of factor manipulation as baseline. The best fit model used random intercepts and slopes of both fixed factors for subjects, and neither random slopes nor intercepts for item. The model reveals a significant interaction of manipulation and context, as well as a significant effect for manipulation, but no effect for context alone, cf. Table 5. According to the hypothesis, a higher H^*^ accent realization is an effective cue for contrastiveness due to the significant interaction. Higher F0 peaks were expected to be congruent in contrastive contexts, and lower F0 peaks in broad focus contexts independent of stimulus origin.

**Table 5:** **Report of the linear mixed effects model with the fixed factors context and manipulation and with congruent/incongruent ratings as dependent variable**.

	**Coefficients**	**SE**	***z*-value**	**Sign**.	**Pr(> |*z*|)**
(Intercept)	0.8907	0.1625	5.482	^*^	0.00001
context = *CF*	−0.3338	0.3401	−0.982	n.s.	0.32629
manipulation = *step5*	0.9754	0.2031	4.804	^*^	0.00001
Interaction	0.9477	0.2747	3.450	^*^	0.00056

* *indicates significance at level p < 0.05, n.s. refers to non significance*.

**Figure 6:**
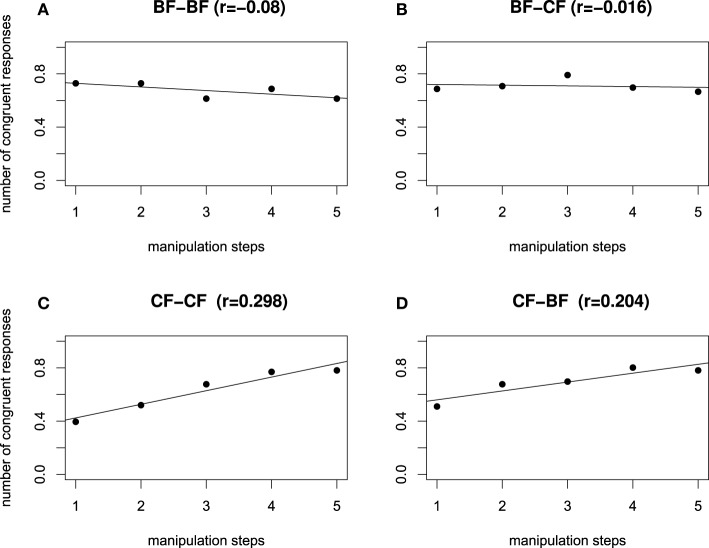
Influence of H^*^ manipulation on the number of congruent responses, separated by dialog type, for all manipulation steps, starting from left to right with step 1 = low H^*^ to 5 = high H^*^. Congruent dialog types BF-BF **(A)** and CF-CF **(C)**, incongruent dialog types BF-CF **(B)** and CF-BF **(D)**.

We computed a Pearson product-moment correlation coefficient to assess the relationship between the manipulation steps and the congruency ratings, separately for each dialog type. Figures 6C,D show that in the contrastive focus context dialogs, a positive correlation between dialog type and manipulation step is evident: an increasing value of the H^*^ peak (manipulation step 1 = low H^*^ value; manipulation step 5 = high H^*^ value), raises the number of congruent responses (CF–CF: *r* = 0.298, CF–BF: *r* = 0.204). On the other hand, in all broad focus context dialogs (Figures 6A,B), the H^*^ peak manipulation does not influence the rating. There was a close to zero correlation between manipulation step and congruency rating (BF–BF: *r* = −0.08, BF–CF: *r* = −0.016). Congruent responses remain at an equal high level, independently of the height of the H^*^ accent.

#### 3.2.5. Discussion

The results reveal two major aspects. First, the manipulation of the pitch peak has a significant effect on the interpretation of the pitch accent. The higher the peak the more often were stimuli rated as congruent in the contrastive focus context. This result was independent of stimulus origin, i.e., whether a stimulus was originally uttered in a broad or contrastive context did not affect its interpretation. It is thus the F0 height (in relation to the previous pitch accents) that caused the perception of contrastiveness in the experiment. This result is in line with previous findings and assumptions on the relationship between contrastive focus and its prosodic realization in German (Bannert, 1985; Alter et al., 2001; Braun, 2005, 2006; Baumann et al., 2006, 2007; Féry and Kügler, 2008; Grice et al., 2009; Sudhoff, 2010).

Second, the obtained significant effect for manipulation points to the fact that the two contexts allow a different amount of prosodic variation. In contrastive contexts, it was clearly the peak manipulation that mattered, and hence, only a certain amount of variation regarding pitch peak scaling was tolerated by listeners. In broad focus contexts, however, listeners accepted both, lower and higher F0 peaks as congruent prosodic realizations, again, independent of stimulus origin. This perceptual behavior mirrors the free variation found in the production of German broad focus contours: Féry and Kügler (2008) showed that downstepped and upstepped pitch accents occur equally frequent (45.7–54.3%) in broad focus contexts. Downstep and upstep correspond in our experiment to the manipulation of the pitch peak, lower scaling refers to downstep, higher scaling to upstep in relation to the prenuclear accent (cf. Figure 4).

Given the significant interaction of manipulation and context we can conclude that the higher scaling of the H^*^ accent reflects a perceptual interpretation of contrastiveness. The next experiment examines whether a manipulation of the low turning point prior to the H^*^ peak can be attributed to a perceptual interpretation of contrastiveness as well, as postulated by Grice et al. (2005).

### 3.3. Perception experiment 3: manipulation of the low turning point

#### 3.3.1. Material

**Figure 7:**
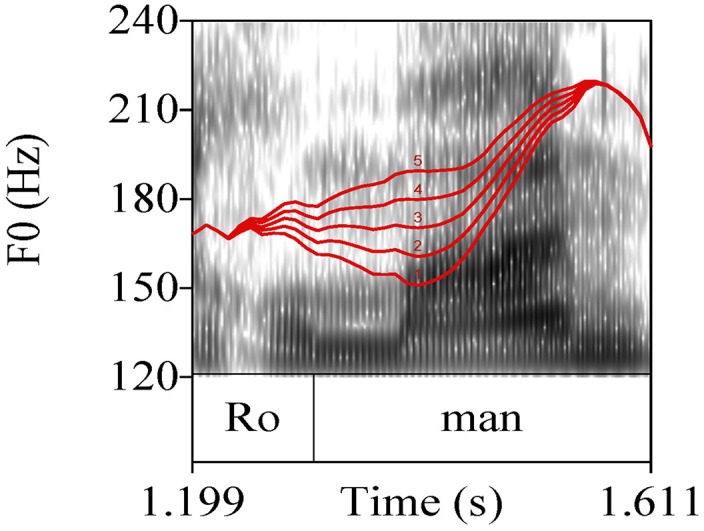
**Illustration of the low turning point manipulation**. Numbers 1–5 illustrate the manipulation steps from the lowest turning point (step 1) to the highest turning point (step 5).

This experiment investigates the role of the low turning point in F0 of the nuclear rise-fall contour, more specifically the issue whether the height of the low turning point interacts with the contrastivity of the context. The sentences for the low turning point manipulation were the same as the ones used for perception Experiment 2. Each sentence was manipulated at the position of the low turning point, cf. Figure 7.

Using a Praat script, manipulation procedure was as follows: The F0 contour of the original file was stylized. The F0 points at the onset of the target word and at the accentual peak were retained and the F0 points between them were deleted. At the time of the label of the low turning point (see production study) a pitch point was inserted, and pitch was interpolated between the remaining pitch points. The end points of the F0 height continuum of the inserted pitch points were determined relative to the F0 height that was produced in the utterance. A distance of two standard deviations from the mean in both directions resulted in a manipulation range from 150 to 190 Hz for each sentence. Thus, five stimuli with a difference of 10 Hz between the low turning points were created, cf. Figure 7.

Each manipulated target sentence was concatenated with an originally congruent context question (BF–BF, CF–CF) and with an originally incongruent context question (CF–BF, BF–CF), resulting in a total of 80 target sentences (4 sentences × 2 focus conditions × 2 contexts × 5 manipulations). These 80 target sentences were scaled at an intensity of 70 db, and stimuli were subdivided into two lists of 40 stimuli each (see the Supplementary Material for the stimuli and their groupings). The experimental task was identical to that one of perception Experiment 2. The experiment lasted approximately 15 min.

#### 3.3.2. Participants

Forty-eight undergraduate students from Potsdam University (16 male, 32 female) with no hearing deficits took part in this perception experiment. They did not take part in the first or second perception experiment. They were all in their twenties, and were either paid for participation or received course credit points. Participants were divided into two groups to listen to either the first or the second experimental set.

#### 3.3.3. Hypothesis

As in the previous experiment, we predict a significant interaction of the factors manipulation and context based on the assumption that the low turning point in F0 interacts with the contrastivity of the context. We expect a lower F0 turning point to signal contrast, cf. the difference of the schematic contours in (2). The prediction thus is that independent of stimulus origin (originally uttered in a broad or a contrastive context), lower F0 turning points should cause significantly more congruent answers in contrastive contexts. Similarly, higher F0 turning points prior to the accentual peak should cause significantly more congruent answers in broad focus contexts.

#### 3.3.4. Results

**Figure 8:**
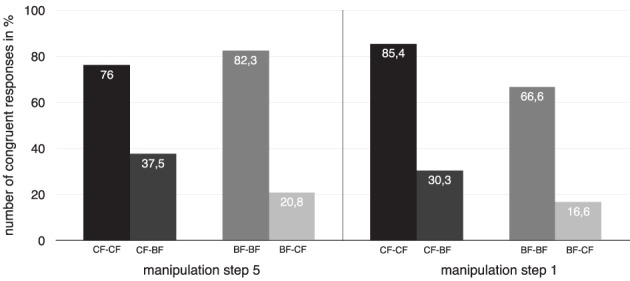
**Number of congruent responses to all dialog types, separated by manipulation step 5–highest low turning point (left-hand bars) and manipulation step 1–lowest low turning point (right-hand bars); black and dark gray bars indicate dialog pairs with contrastive contexts, lighter gray bars indicate dialog pairs with broad focus contexts**.

Figure 8 depicts the number of congruent responses to all dialog types, separated for the highest low turning point manipulation step 5 (left-hand bars) and the lowest low turning point manipulation step 1 (right-hand bars). Independent of manipulation step, congruent context-target dialogs (CF–CF, BF–BF) obtained an equal high number of congruency ratings, while incongruent context-target dialogs (CF–BF, BF–CF) obtained an equal low number of congruency ratings.

As described for perception Experiment 1, Section 3.1.4, we fit a multilevel model with context (with levels BF/CF) and manipulation (with levels step1/step5, i.e., the endpoints of the manipulation range) as fixed factors, and calculated likelihood ratio tests on the basis of backward modeling of the random factors to identify the best fit model. As before, only the endpoints of the manipulation range entered the analysis. Treatment-coding was applied using level *BF* of factor context, and level *step1* of factor manipulation as baseline. The best fit model used crossed random factors participant and item, applying random slopes and intercepts for both fixed factors with participants, and random slopes with item for the fixed factor context. The model reveals no significant interaction, and no significant effect of the fixed factors context and manipulation, cf. Table 6. According to our hypothesis, the factor manipulation was defined such that the lowest manipulation step should result in a contrastive interpretation. Thus, the lowest manipulation step was expected to be rated more congruent in contexts that require a contrastive interpretation in the answer. Consequently, the highest manipulation step should result in a non-contrastive interpretation, thus should be rated more congruent in contexts that require a non-contrastive interpretation of the answer.

**Table 6:** **Report of the linear mixed effects model with the fixed factors context and manipulation and with congruent/incongruent ratings as dependent variable**.

	**Coefficients**	**SE**	***z*-value**	**Sign**.	**Pr(> |*z*|)**
(Intercept)	0.0888	0.1736	0.512	n.s.	0.6089
context = *CF*	0.4521	0.2688	1.682	n.s.	0.0926
manipulation = *step5*	0.1771	0.1479	1.198	n.s.	0.2311
Interaction	−0.2227	0.1479	−1.506	n.s.	0.1321

*n.s. refers to non significance*.

**Figure 9:**
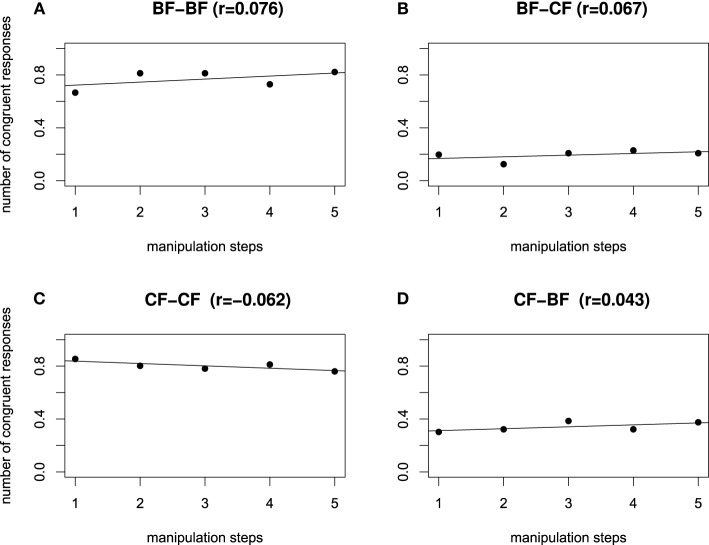
**Influence of L manipulation on the number of congruent responses, separated by dialog type, for all manipulation steps, starting from left to right with step 1: low L (150 Hz), step 2: (160 Hz), step 3: (170 Hz), step 4: (180 Hz), step 5: high L (190 Hz)**. Congruent dialog types BF-BF **(A)** and CF-CF **(C)**, incongruent dialog types BF-CF **(B)** and CF-BF **(D)**.

As for Experiment 2, we computed a Pearson product-moment correlation coefficient to assess the relationship between the manipulation steps and the congruency ratings, separately for each dialog type. Figure 9 shows no correlation between manipulation step and congruency ratings for either of the dialog pairs. In other words, the close to zero correlations show that the manipulation had no influence on the congruency rating, which is in line with the non-significant interaction of the factors context and manipulation, cf. Table 6. However, Figure 9 shows a difference in level of congruency ratings, i.e., congruent dialogs were rated more congruent (cf. Figures 9A,C) than incongruent ones (cf. Figures 9B,D).

#### 3.3.5. Discussion

The results of the manipulation of the low F0 turning point reveal two aspects. First, independently of the prosodic manipulation, congruent context-target dialogs were rated better than incongruent dialogs. Second, the non-significant interaction of manipulation and context suggest that the low turning point before the accentual peak does not contribute to the perceptive impression of contrast. If it would, it was expected that the number of congruency ratings for manipulations CF–CF:5 (190 Hz) and BF–BF:1 (150 Hz) would have been considerably lower, likewise the number of congruency ratings for manipulations BF–CF:1 (150 Hz) and CF–BF:5 (190 Hz) would have been higher. Taken the results of the H^*^ manipulation from the previous experiment together with the results of this experiment suggest that the higher scaling of the H^*^ accent stimuli is the relevant cue that signals contrastivity perceptually in German.

## 4. Discussion and conclusion

This study was concerned with the phonetics of the nuclear rise-fall contour in German. In particular, we investigated how the phonetic realization of the rise-fall contour interacts with contexts that require a contrastive or broad focus interpretation in the answer. To this end, a production experiment and a series of perception experiments were carried out. The analysis of the production data revealed that contrastive focus changes the phonetics of the rise-fall contour. Speakers realized significantly higher and later F0 peaks in contrastive contexts. The realization of the low turning point prior to the accentual peak showed no significant differences. The fact that contrastive focus raises nuclear H^*^ accents in German confirms earlier results (Baumann et al., 2006, 2007; Féry and Kügler, 2008; Grice et al., 2009).

A series of semantic congruency experiments investigated the perceptual role of the phonetic differences found in the production experiment. The first perception experiment investigated whether listeners were able to perceive the phonetic differences found in production as a function of focus using congruent (BF–BF and CF–CF) and incongruent dialogs (BF–CF and CF–BF). Interestingly, the results of the perception study show that listeners are able to distinguish between congruent and incongruent dialogs, (see Figure 3) although the acoustic differences reported in Table 2 were small. This might reveal that the overall shape of the intonation contour involves cues to perceive a contrastive or non-contrastive interpretation of an answer. As was shown in Table 3, prenuclear pitch accents in sentences containing a contrastive focus were realized lower on average before nuclear accents, while they were higher on average in case of broad focus sentences. This relation between the height of prenuclear and nuclear pitch accents seems to point to the fact that a nuclear rise-fall contour may be interpreted more global rather than locally at the nuclear pitch accent.

In order to investigate which parts of the rise-fall contour functionally interact with a contrastive interpretation, two separate perception experiments were conducted that examined whether the higher scaling of H^*^ accents causes the perceptual impression of contrastive focus, or whether the lower scaling of the low turning point is a sufficient phonetic cue. To this end, sentences with manipulated height values of the H^*^ peak, and of the low turning point were generated, respectively. The perception of the H^*^ accent manipulation revealed that a higher scaling of the H^*^ accent increased the perceptual impression of a contrastive accent. Specifically, contrastive contexts required higher F0 values. Broad focus context allowed both, lower and higher H^*^ values, see (Féry and Kügler, 2008) for similar variations in speech production. Consequently, the free variation of upstepped accents (Féry and Kügler, 2008) and downstepped accents (Féry, 1993; Féry and Kügler, 2008; Grice et al., 2009) in broad focus contexts in speech production mirrors speech perception. The manipulation of the low F0 turning point, in turn, did not show an indication of a contrastive interpretation since the number of congruent responses did not change as a function of the low turning point value. The results appear to support the assumption that a contrastive focus compared to a broad sentence focus does not cause a different phonological category in German, but speak in favor of an interpretation that focus affects the pitch register (Féry and Kügler, 2008; Féry and Ishihara, 2010).

### 4.1. The on-ramp vs. off-ramp debate

The experiments presented in this paper are partly related to the debate of how to analyse pitch accents, the so called “on-ramp” vs. “off-ramp” approach (Gussenhoven, 2004). The crucial assumption in the “off-ramp” approach is that the F0 movement **from** the pitch target is the essential of the pitch accent (off-ramp), whereas the “on-ramp” approach analyzes the F0 movement **toward** a pitch target as belonging to the pitch accent (on-ramp). The on-ramp approach is grounded in the ToBI tradition, which is “a system for transcribing the intonation patterns and other aspects of the prosody” of spoken utterances in a language variety (Beckman and Ayers-Elam, 1997). The off-ramp approach was initiated by Gussenhoven (1984) and particularly studied in Hanssen et al. (2008) and Chen (2011).

Related to the present study, a rise-fall contour is phonologically analyzed as L+H^*^ L− in the “on-ramp” approach (Grice et al., 2005). The GToBI guidelines suggest to interpret a low turning point in F0 prior to the rise toward the accentual peak as a tone, while the perceptual impression of the stressed syllable is high (or rising). Hence, the rise is phonologically interpreted as a result of an F0 transition between a low leading tone (L+) and the accentual high tone (H^*^) [cf. (2-a)].

From the off-ramp perspective, a rise-fall contour is analyzed as a phonological fall H^*^+L following a phonetic rise (Féry, 1993; Grabe, 1998; Peters, 2014). The rise may vary in steepness and shape, but crucially it is not phonologically interpreted by means of a tone. With respect to the alignment of falling H^*^+L pitch accents in German (Grabe, 1998) found that, in general, the position of the accentual peak is at the right edge of the accented syllable's rime. Hence, there is an F0 transition toward the accentual peak, which however, is interpreted as a phonetic onglide that does not necessarily rise, or whose steepness may vary. Grabe (1998) carefully distinguished between non-final and final nuclear falling accents. Only in case of final falling accents, which are realized on a phrase-final accented syllable (e.g., [ˈvɔlf] in *Ich bin der Wolf*. “I'm the wolf.” p. 73f), the peak position is realized earlier, that is at the onset of the accented vowel. This structural dependent variation of the accentual F0 peak led Grabe to conclude that the onglide only has phonetic properties since the onglide is less elaborated in the case of phrase-final accented syllables.

Similarly, structural conditions were found as evidence for an off-ramp analysis of Dutch prenuclear falling accents (Chen, 2011). In a comparison of prenuclear high and falling accents Chen (2011) observed a structural distinction rather than a functional one: independent of the information structural context (topic vs. focus) in which the accents were realized, the amount of sonorant segments within and after the accented syllable determined the accent pattern. If enough sonorant segments were present, a falling accent (H^*^+L) was realized, if less sonorant material was present, a high rise (H^*^) was realized. Similar to Grabe (1998), Chen (2011) concludes that the lack of a functional distinction of the two pitch accent types points to the fact that the distinction is phonetically motivated rather than phonologically determined.

As an alternative to the on-ramp and off-ramp interpretations of tonal contours, there are languages exhibiting tones that do not carry meaning, e.g., the accentual phrase tones in Tokyo Japanese (Gussenhoven, 2004), as opposed to a language like English where all post-lexical tones are supposed to carry meaning (Pierrehumbert and Hirschberg, 1990). On this note, the German rise-fall contour may constitute a case where the scaling of the accentual peak clearly contributes the interpretation of the contour with respect to contrastiveness while the rising part of the rise-fall contour does not contribute to this meaning, as our experiment three showed. Thus, a phonological interpretation of the rise-fall contour as L+H^*^ L− would be similar to the on-ramp approach except that contrary to the assumption proposed in Grice et al. (2005), the leading low tone does not carry meaning.

Along these lines, our perceptual results of the manipulated stimuli may suggest that the onglide toward a high accentual F0 peak is either a phonetic transition (in the sense of the off-ramp approach) or a leading low tone that does not carry meaning. If the rise would have been a reflex of a phonological tone (L+) that carries a contrastive meaning as in English (Pierrehumbert and Hirschberg, 1990) native German listeners were expected to perceive this tone in the corresponding contexts. In particular, we were expecting a functional difference between a L+H^*^ accent and a simple H^*^ accent based on the assumption that L+H^*^ carries the meaning of contrast (Grice et al., 2005) given a similar functional distinction in English intonation (Pierrehumbert and Hirschberg, 1990; Beckman and Ayers-Elam, 1997). Manipulating the scaling of the onset of the rise (perception Experiment 3) did however not reveal that listeners relate a lower scaling to be congruent with a context that elicits contrast. We can thus conclude that a leading low tone does not seem to carry contrastive meaning in German.

### 4.2. Conclusion

This study investigated the phonetics of the rise-fall contour in German. In particular, it was tested whether phonetic differences in the rise-fall contour were realized in relation to contrastive and non-contrastive contexts, and which parts of the rise-fall contour seem to play a functional role in perception. The acoustic analysis of nuclear rise-fall contours elicited in broad and contrastive focus contexts revealed a significant difference for the realization of the accentual high tone, yet not for the low F0 turning point prior to the accentual high. In a series of semantic congruency perception tests, listeners judged the congruency of congruent and incongruent context-stimulus pairs on the basis of three different sets of stimuli: (i) original data from the production study in congruent contexts and cross-spliced yielding incongruent dialogs, (ii) stimuli with manipulated accentual high tone that were combined with originally congruent contexts and, again, cross-spliced with originally incongruent contexts, and (iii) stimuli with manipulated low F0 turning point of the rising part of rising-falling accent shapes, again combined with congruent and incongruent contexts. The first perception experiment revealed that listeners distinguish between nuclear rising-falling contours with respect to their focus context. The second perception experiment revealed that independent of stimulus origin, higher F0 peaks were rated significantly more frequent as congruent to contrastive focus contexts than lower peaks; hence, the scaling of the nuclear peak determined its contextual interpretation in our experiments as assumed in the literature on German intonation (Bannert, 1985; Alter et al., 2001; Braun, 2005, 2006; Baumann et al., 2006, 2007; Féry and Kügler, 2008; Grice et al., 2009; Sudhoff, 2010), and as argued by Gussenhoven (2004) in relation with the interpretation of focus in terms of the effort code. With respect to broad focus contexts, the results show that both upstepped and downstepped contours are rated as equally congruent reflecting a free variation of the realization of the final (nuclear) accent in broad focus in speech production in German (Féry and Kügler, 2008). The third perception experiment revealed that manipulation of the low F0 turning point did not affect the perception as a function of focus context. Stimulus origin was rated more congruent than F0 manipulations.

The results of the perception experiments suggest that the scaling of the accentual peak is sufficient to license a contextual interpretation of a nuclear rising-falling accent shape (perception Experiment 2). The manipulation of a low F0 turning point prior to the accentual peak as a potential reflex of a low leading tone (L+) does not drive the perception as a function of focus context (perception Experiment 3). The results seem to support the view that focus affects the pitch register (Féry and Kügler, 2008; Féry and Ishihara, 2010), in our data a fact of pitch register raising of the nuclear accent peak. The production data also showed that the relation between prenuclear and nuclear accent peaks varies as a function of focus context. If the functional interpretation of pitch accents depends only on their local scaling, or if it is a matter of pitch accent relations within a sentence, or a combination thereof needs to be shown in future research.

## Conflict of interest statement

The authors declare that the research was conducted in the absence of any commercial or financial relationships that could be construed as a potential conflict of interest.
