# Supplementary material for: Production and perception of contrast: The case of the rise-fall contour in German
Source: Front Psychol. 2015 Sep 2;6:1254. doi: 10.3389/fpsyg.2015.01254 (PMC4557098; doi:10.3389/fpsyg.2015.01254)
Supplement: Supplementary file 1 [file DataSheet1.PDF]

Appendix

Speech production experiment: Sentence material

The experimental target sentences had a subject (S), auxillary (Aux), optional adverb (Adv), object (Obj) and verb (V) order. Target words were represented by the object which was either monosyllabic, disyllabic, or trisyllabic. Target words have the following word frequencies based on a dlexDB corpus query (http://www.dlexdb.de/query/kern/lem/list/): Wal—268, Hahn—1901, Roman—5966, Fasan—183, Admiral—3184, General—24064. Different aspects of word frequency on different linguistic levels influence speech production and perception (e.g., Pierrehumbert, 2001; Ellis, 2002; Ernestus, 2014), and recent work indicates that this includes tonal aspects as well (Yang et al., 2013; Schweitzer et al., 2015). We did not control for word frequencies of the target words in our experimental design, and thus, word frequency might be a possible effect influencing the results that we report.

Each sentence was spoken as response to a context question, which either elicited a broad focus or a contrastive focus. A total of 384 target sentences (8 speakers x 2 focus conditions × 6 target words × 4 sentence lengths) had been recorded. The target sentences are listed in the following.

(I) Target word *Wal* 'whale'

a. Martin hat den Wal gesehen.
'Martin has seen the whale.'
b. Martin hat gestern den Wal gesehen.
'Yesterday, Martin has seen the whale.'
c. Martin hat zufälligerweise den Wal gesehen.
'Coincidentally, Martin has seen the whale.'
d. Martin hat gestern zufälligerweise den Wal gesehen.
'Coincidentally, Martin has seen the whale yesterday.'

(II) Target word *Hahn* 'cock'

a. Maja hat den Hahn gefüttert.
'Maja has fed the cock.'
b. Maja hat gestern den Hahn gefüttert.
'Yesterday, Maja has fed the cock.'
c. Maja hat zufälligerweise den Hahn gefüttert.
'Coincidentally, Maja has fed the cock.'
d. Maja hat gestern zufälligerweise den Hahn gefüttert.
'Coincidentally, Maja has fed the cock yesterday.'

(III) Target word *Roman* 'novel'

a. Martin hat den Roman gelesen.
'Martin has read the novel.'
b. Martin hat gestern den Roman gelesen.
'Yesterday, Martin has read the novel.'
c. Martin hat zufälligerweise den Roman gelesen.
'Coincidentally, Martin has read the novel.'
d. Martin hat gestern zufälligerweise den Roman gelesen.
'Coincidentally, Martin has read the novel yesterday.'

(IV) Target word *Fasan* 'pheasant'

a. Maja hat den Fasan gehört.
'Maja has heard the pheasant.'
b. Maja hat gestern den Fasan gehört.
'Yesterday, Maja has heard the pheasant.'
c. Maja hat zufälligerweise den Fasan gehört.
'Coincidentally, Maja has heard the pheasant.'
d. Maja hat gestern zufälligerweise den Fasan gehört.
'Coincidentally, Maja has heard the pheasant yesterday.'

(V) Target word *Admiral* 'admiral'

a. Martin hat den Admiral gegrüßt.
'Martin greeted the admiral.'
b. Martin hat gestern den Admiral gegrüßt.
'Yesterday, Martin greeted the admiral.'
c. Martin hat zufälligerweise den Admiral gegrüßt.
'Coincidentally, Martin greeted the admiral.'
d. Martin hat gestern zufälligerweise den Admiral gegrüßt.
'Coincidentally, Martin greeted the admiral yesterday.'

(VI) Target word *General* 'general'

a. Maja hat den General getroffen.
'Maja has met the general.'
b. Maja hat gestern den General getroffen.
'Yesterday, Maja has met the general.'
c. Maja hat zufälligerweise den General getroffen.
'Coincidentally, Maja has met the general.'
d. Maja hat gestern zufälligerweise den General getroffen.
'Coincidentally, Maja has met the general yesterday.'

Perception Experiment 2: Manipulation of the low turning point

Distribution of dialogs per group. Each dialog is composed of a context, either a contrastive focus context (CF) or a broad focus context (BF), and a target sentence, which was either originally realized under contrastive focus (CF) or under broad focus (BF). Context information is represented by the first position of the dialog name, target information is represented by the second position. Numbers after each dialog present the height of the f0 turning point , i.e., 1 displays the lowest f0 value of the low turning point (150 Hz), 5 displays the highest f0 value of the low turning point (190 Hz).

Group 1: congruent context-answer pairs
BF-BF Roman: 1, 2, 3, 4, 5
BF-BF Admiral: 1, 2, 3, 4, 5
CF-CF Fasan: 1, 2, 3, 4, 5
CF-CF General: 1, 2, 3, 4, 5

Group 1: incongruent context-answer pairs
CF-BF Roman: 1, 2, 3, 4, 5
CF-BF Admiral: 1, 2, 3, 4, 5
BF-CF Fasan: 1, 2, 3, 4, 5
BF-CF General: 1, 2, 3, 4, 5

Group 2: congruent context-answer pairs
BF-BF Fasan: 1, 2, 3, 4, 5
BF-BF General: 1, 2, 3, 4, 5
CF-CF Roman: 1, 2, 3, 4, 5
CF-CF Admiral: 1, 2, 3, 4, 5

Group 2: incongruent context-answer pairs
CF-BF Fasan: 1, 2, 3, 4, 5
CF-BF General: 1, 2, 3, 4, 5
BF-CF Roman: 1, 2, 3, 4, 5
BF-CF Admiral: 1, 2, 3, 4, 5

Perception Experiment 3: Manipulation of the H* accent

Distribution of target sentences per group. Manipulation was performed individually for each sentence in relation to its prenuclear accent. 1 displays the lowest H* value, 5 displays the highest H* value. Lowest H* value (min) and highest H* value (max) were computed for each sentence as follows:
min = F0 (prenuclear accent) −30 Hz
max = F0 (prenuclear accent) +50 Hz

Group 1: congruent context-answer pairs
BF-BF Roman: 1, 2, 3, 4, 5 (1:203, 5:283), value of the prenuclear accent: 233 Hz
BF-BF Admiral: 1, 2, 3, 4, 5 (1:204, 5:284), value of the prenuclear accent: 234 Hz
CF-CF Fasan: 1, 2, 3, 4, 5 (1:170, 5:250), value of the prenuclear accent: 200 Hz
CF-CF General: 1, 2, 3, 4, 5 (1:171, 5:251), value of the prenuclear accent: 201 Hz

Group 1: incongruent context-answer pairs
CF-BF Roman: 1, 2, 3, 4, 5 (1:203, 5:283), value of the prenuclear accent: 233 Hz
CF-BF Admiral: 1, 2, 3, 4, 5 (1:204, 5:284), value of the prenuclear accent: 234 Hz
BF-CF Fasan: 1, 2, 3, 4, 5 (1:170, 5:250), value of the prenuclear accent: 200 Hz
BF-CF General: 1, 2, 3, 4, 5 (1:171, 5:251), value of the prenuclear accent: 201 Hz

Group 2: congruent context-answer pairs
BF-BF Fasan: 1, 2, 3, 4, 5 (1:196, 5:276), value of the prenuclear accent: 226 Hz
BF-BF General: 1, 2, 3, 4, 5 (1:220, 5:300), value of the prenuclear accent: 250 Hz
CF-CF Roman: 1, 2, 3, 4, 5 (1:208, 5:288), value of the prenuclear accent: 238 Hz
CF-CF Admiral: 1, 2, 3, 4, 5 (1:157, 5:237), value of the prenuclear accent: 187 Hz

Group 2: incongruent context-answer pairs
CF-BF Fasan: 1, 2, 3, 4, 5 (1:196, 5:276), value of the prenuclear accent: 226 Hz
CF-BF General: 1, 2, 3, 4, 5 (1:220, 5:300), value of the prenuclear accent: 250 Hz
BF-CF Roman: 1, 2, 3, 4, 5 (1:208, 5:288), value of the prenuclear accent: 238 Hz
BF-CF Admiral: 1, 2, 3, 4, 5 (1:157, 5:237), value of the prenuclear accent: 187 Hz
